# Dietary practice and associated factors among lactating mothers in Dangila District in the Awi Zone Amhara region Ethiopia, 2022: a cross-sectional study

**DOI:** 10.3389/fnut.2024.1506707

**Published:** 2025-01-06

**Authors:** Sileshi Mulatu, Getasew Mulatu, Azeb Gedif

**Affiliations:** ^1^Department of Pediatrics and Child Health Nursing, College of Medicine and Health Science, Bahir Dar University, Bahir Dar, Ethiopia; ^2^Department of Emergency and Critical Care Nursing, College of Medicine and Health Science, Bahir Dar University, Bahir Dar, Ethiopia

**Keywords:** lactating mothers, dietary practice, dietary diversity, food security, Dangila, Ethiopia

## Abstract

**Introduction:**

Lactation is the critical time for meeting the nutritional demands of mothers and infants. Mothers from low-income countries, including Ethiopia, are considered nutritionally vulnerable. Good dietary practices for mothers during lactation are therefore important for the good health of their children. The main objective of this study was to assess dietary practice and associated factors of lactating mothers in Dangila Zuria District, Amhara region, Ethiopia, 2022.

**Methods:**

A community-based cross-sectional study was conducted among 609 lactating women from March 1st to May 1st 2022. The study participants were selected using a straightforward simple random sampling techniques. Data was collected by using structured questionnaires. The data was entered in Epi-data and exported to SPSS version 23 for analysis. Bi-variable and multi-variable logistic regression analyses were used to see the relevant associations. The variable significantly associated with dietary practice was identified based on a *P*-value ≤ 0.05.

**Results:**

This study revealed that only one-third 204 lactating mothers, demonstrated good dietary practices. Notably, several factors were significantly associated with poor dietary practices: mothers with a family size greater than four had 3.01 times higher odds of inadequate dietary habits (AOR = 3.01; 95% CI: 1.56, 9.28), while those with birth intervals of <2 years faced 2.31 times greater odds (AOR = 2.31; 95% CI: 1.49, 3.56). Additionally, daily laborers exhibited a striking 9.35 times higher likelihood of poor dietary practices (AOR = 9.35; 95% CI: 8.02, 19.96), and food-insecure mothers had 4.93 times higher odds of inadequate nutrition (AOR = 4.93; 95% CI: 3.40, 7.16).

**Conclusion:**

In conclusion, the dietary practices of lactating mothers were found to be suboptimal, falling below WHO and FAO recommendations. Factors such as family size, birth intervals, maternal occupation (especially daily laborers), and food insecurity were identified as significant contributors. Addressing these issues is crucial. Key stakeholders, including the Ministry of Health (MOH), Regional Health Bureaus (RHB), and health professionals, must work together to alleviate food insecurity, promote optimal family planning, and educate mothers on proper nutrition. Addressing these challenges is essential to achieving global objectives such as reducing malnutrition, improving maternal health, and ensuring healthy child development.

## 1 Introduction

### 1.1 Background

The lactation period refers to the time during which the mammalian produces milk to feed their young (1). Lactation itself is the physiological process of milk production, which is stimulated by hormones such as prolactin and oxytocin (2). During the lactation time, the mother's body changes to produce milk in response to the baby's suckling. Proper nutrition and hydration are required to maintain the milk supply of the mothers. Access to good dietary practices is fundamental to the survival of lactating mothers as well as the newborn's good growth and development (1). Maternal health during lactation is crucial, not just for individual women but also for their newborns and families (3). In low and middle-income countries like Ethiopia, women of reproductive age often face nutritional vulnerabilities that can adversely affect their wellbeing and that of their newborns (4). Lactating mothers' dietary practices are influenced by a variety of factors, including cultural beliefs, socio-economic conditions, and access to healthcare, with significant global variation (5). While international guidelines recommend specific nutritional intake for lactating women, adherence to these recommendations often varies due to economic constraints, lack of education, and limited access to healthcare, especially in low-resource settings (6, 7). There are gaps in research regarding the impact of culturally specific dietary practices, the effectiveness of nutritional interventions, and the long-term effects of maternal diet on both maternal and infant health (8).

Good dietary practice is not simply two or more additional feedings than usual (9), but it includes a variety of food groups in the daily patterns, and women in low and middle-income countries, especially in our country Ethiopia, have a limited intake of animal sources foods, fruits, and vegetables, and their intake of micronutrients is less than the recommended values, which can increase women's risk of micronutrient deficiencies and their child's for poor growth and development with inadequate breast milk (10, 11). In general, good nutrition (good dietary practice) during pregnancy and lactation is the cornerstone for the survival, health, and development of current and succeeding generations. However, almost more than half of the lactating mothers do not consume the recommended dietary practice during this period and they face protein-energy malnutrition, nutritional anemia, vitamin A deficiency, and iodine deficiency disorders (IDD) (12, 13).

Dietary practices before and during pregnancy and lactation periods have been shown to induce long-term effects on the later health of the child, including the risk of common non-communicable diseases such as obesity, diabetes, and cardiovascular disease (14). Poor dietary practices and poor health from preventable causes disproportionately affect the wellbeing of millions of people in the developing world.

Poor dietary practices affect over 850 million people worldwide and 200 million adults in Sub-Saharan Africa (SSA), especially affecting children and their mothers Worldwide (15, 16).

More than 3.5 million women and children under the age of five in developing countries die each year due to the underlying cause of under-nutrition which is caused by poor dietary practices (17, 18). Studies show that lactating women from developing countries are vulnerable to poor dietary practices. This is true in our country: poor dietary practice is the leading cause of maternal and child malnutrition, which leads to poor secretion of nutrients in breast milk, and this can have a long-term impact on the child's growth and development (19–21). In general, to improve maternal health and their child's growth and development, it is important to know the burden of poor dietary practice and its associated factors. A study conducted in Tigray, Moyale District, Borena Zone, and Gondar showed that most lactating mother's dietary practices are below WHO and FAO recommendations (8, 22–24).

Despite the efforts of the government to improve nutrition, few studies done on maternal nutrition in the country revealed sub-optimal nutrient intakes of pregnant lactating women compared with the standard recommended by the WHO (25, 26). Only about few lactating women who attend antenatal care at public hospitals reported good dietary practices (27). Similarly, studies also identified education, monthly income, attitude, and gravidity as factors associated with the dietary practices of lactating women (28, 29).

According to current evidence, exclusive breastfeeding mothers are advised to consume an additional 300–500 calories per day or include 1–2 extra snacks to meet the increased nutritional demands of lactation, which are significantly higher than those during pregnancy or for non-pregnant women. However, in Ethiopia, many lactating mothers lack training or awareness about the importance of increasing meal frequency during this period (30, 31). The challenge to ensure consistent quality dietary practice for lactating mothers in Ethiopia is because half of the population is living below the food poverty line and cannot meet their daily minimum nutritional requirement (20, 32). Taking this into consideration, the Ethiopian government continuously showed its commitment to averting nutritional problems by developing food and nutrition policies, strategies, and programs targeted at improving the nutritional status of pregnant and lactating women and interventions had been going on through the routine health care services (33). Therefore, this study aimed to assess the dietary practices and associated factors among lactating women in the Awi Zone Dangila Zuria Woreda.

## 2 Methods

### 2.1 Study area and design

A community-based cross-sectional study was employed to assess dietary practice and associated factors among lactating mothers in Dangila Zuria Woreda, Awi Zone Ethiopia. The study was conducted in Dangila Zuria Woreda Northwest Ethiopia, from March 1st to May 1st in 2022.

Dangila Zuria Woreda is one of the eleventh Woreda in the Awi Zone of Amhara Regional State, which is located 485 km away from the capital, Addis Ababa, in the North West Ethiopia, and 78 km away from Bahir Dar the capital city of the region and 40 kms from the Zonal Capital city Injibara. Administratively, the district is structured into six clusters and according to the Ethiopian statistical service the population size of towns by sex, region, zone and woreda as of July 2021, the district, has a total population of 53,225 out of which 25,813 are females and 27,412 are males. The estimated number of lactating mothers in the district is 3,520. The district census which was obtained from the Woreda health office used as a baseline. The Woreda currently has one district hospital & 6 health centers, and 27 health posts. The major agricultural products are Teff, Dagussa, Maize, Potato, Sorghum, peas and beans, and some vegetables like cabbage, carrots tomatoes.

### 2.2 Source and study population

The source population for this study comprised all lactating mothers residing in the Dangila Zuria Woreda, with the study population specifically including those living in selected kebeles. The study unit consisted of mothers who were selected as samples and interviewed. Lactating mothers paired with a child under 2 years old who had lived in the Woreda for at least 6 months were included in the study, while lactating mothers who were critically ill and those whose children with critical illnesses were excluded from participation in the study.

### 2.3 Sample size determination

A single population proportion formula was used to determine the sample size, allowing for the estimation of a sufficient number of participants. The sample was calculated for both the dependent and independent variables taking the prevalence of poor dietary practices among lactating mothers as 71.2% for lactating mothers (8), by considering 5% marginal error, 95% CI, and 2 as the design effect, Based on this, the actual sample size were calculated using the formula for single population proportion.

Therefore;


$$\begin{array}{l} n=\frac{z^{2}p\left(1-p\right)}{d^{2}} \end{array}$$

Where Z = level of confidence at (1.96)^2^ 95% CI

P = Single Population Proportion (71.2%).

d = Margin of error 5% (0.05).

n = Sample Size


$$\begin{array}{l} \text{Calculation }n=\frac{{\left(1.96\right)}^{2}\left(0.712\left(1-0.712\right)\right)}{0.0025}=\;316 \end{array}$$

When we considered the design effect of 2 for the assumption of multistage sampling, across the clusters, the total sample was 316 × 2 = 632.

### 2.4 Sampling procedures

We employed a multi-stage sampling technique to systematically select kebeles, households, and lactating mothers for our study. First, we focused on the Dangila District, which comprises 27 rural kebeles. To ensure a representative sample, we randomly selected six kebeles from the total kebeles using a lottery method, which helps eliminate bias in the selection process.

Once the kebeles were chosen, we allocated lactating mothers to each kebele based on the population size of the kebele, ensuring the representation was proportional and used to capture a diverse range of experiences and perspectives across different community sizes. Within each selected kebele, we then identified individual lactating mothers using simple random sampling.

### 2.5 Operational definition

**Dietary practice:** is an observable action or behavior of eating habits, food choices, and nutritional behaviors of women who are breastfeeding.

**Poor dietary practice**: lactating women who score below 50% for dietary practice-related questions in 24 h. Unhealthy eating habits can include inadequate caloric and nutrient intake (11).

**Good dietary practice**: lactating women who score above 50% for dietary practice-related questions in 24 h (11).

**Adequate dietary diversity:** refers to households consuming at least four different food groups within the 24 h before the survey.

**Inadequate dietary diversity:** when households consumed less than four food groups within 24 h before the survey.

**Food insecurity:** the limited or uncertain access to sufficient and nutritious food among lactating mothers, insufficient availability of food in the household, or disruptions in food supply.

### 2.6 Data collection procedures

A structured questionnaire that was given by an interviewer was used to collect data, which made it easier to compile information in an organized manner. Which comprised; socio-demographic characteristics, source of food, feeding practice and other individual-related factors; dietary diversity, and food security-related questions.

The questionnaire designed to assess the eating habits of lactating mothers was adapted from existing studies to better suit our local context. We included 11 questions to evaluate the dietary practices of lactating mothers. The dietary practice was assessed by using open-ended surveys such as dietary recalls or records, or using closed-ended surveys including food frequency questionnaires and we assessed the dietary practice by using the modified form of the nine-item scales taken from previous literature (23, 34). The items were focused on the short-term dietary practices of lactating mothers. The score of the respondents was taken and respondents were classified as having good or poor dietary practices by taking their responses ≥50% and <50%, respectively (8, 32).

The items had “Yes” or “No” responses. Lactating mothers who scored ≥50% for the responses were classified as having good dietary practice and poor dietary practice otherwise. The rest questioner was adapted and modified from WHO and other similar studies (8, 35). Five diploma nurses' data collectors and two BSc nurse supervisors participated in the data collection. The 2-day training was given to the data collectors and supervisors on the approach of data collection.

### 2.7 Data quality control

To ensure the integrity and accuracy of the data, all data collectors and supervisors underwent comprehensive orientation and training on the processes for reviewing and recording information, which aimed to minimize potential biases during the data collection. A pretest was conducted in Fagita Lokoma Woreda with a sample of 20 lactating mothers to evaluate the consistency and completeness of the data collection tools, allowing us to identify and address any issues before the main data collection commenced.

During the data collection period, close supervision and monitoring were implemented by both supervisors and the perinatal investigator. This involved regular check-ins and feedback sessions to reinforce proper data collection techniques and address any challenges that arose in real-time. After data collection, a thorough review was conducted by both the supervisor and the investigator to check for completeness and accuracy.

Any discrepancies or missing data were addressed promptly. Furthermore, data entry procedures were standardized, and validation checks were applied to identify potential errors.

### 2.8 Data processing and analysis

The data was coded and entered Epi-data then exported to SPSS software package version 23 for analysis. Percentages, frequency distributions, proportions, mean and range were done and used for describing descriptive data. Then bi-variable logistic regression was made to see the crude significant relation of each independent variable with dependent variables.

Finally, independent variables with *P*-value < 0.2 were entered into a multi-variable logistic regression to control the effect of confounding. An odds ratio with a 95% confidence interval to ascertain the association between the independent and dependent variables was used. A *p*-value of less than 5% was used to declare the association between factors and the dependent variable.

### 2.9 Ethical approval

The research was approved by the Institutional Review Board (IRB) at Bahir Dar University College of Medicine and Health Sciences. Ethical approval was granted on 6 September 2021, under protocol number 280/2021. The IRB thoroughly reviewed the study design to ensure it adhered to ethical standards, safeguarding the rights and wellbeing of all participants. All experiments were conducted in strict accordance with relevant national and international ethical guidelines, including the Declaration of Helsinki and the Ethiopian National Guidelines for Biomedical Research. These guidelines were followed to minimize harm to participants and maintain the integrity of the research. Informed consent was obtained from all participants prior to the start of the study. Participants were provided with detailed information about the study's objectives, procedures, potential risks, and their rights. Written informed consent was obtained from all participants, ensuring their participation was voluntary and based on a clear understanding of the study.

## 3 Results

### 3.1 Socio-demographic characteristics

In this comprehensive study of 609 lactating mothers aged 15–49 years, an impressive response rate of 96.36% was recorded. A significant majority, of lactating mothers (79.8%) identified as part of the Amhara ethnic group. Nearly half (47.8%) of lactating mothers were aged 25–34 years, and a substantial 61.9% were unable to read or write.

Most participants (77.8%) of lactating mothers were housewives in their occupational status, while over two-thirds (69.8%) of their husbands were engaged in farming and nearly half (47.6%) of the husbands were also illiterate. Financially, a large portion of the lactating mothers (88.2%) reported a monthly income between 1,000 and 2,000 Ethiopian Birr, and more than two-thirds (65.2%) lived in households with 4–6 family members. Of the total two-thirds (64.9%) of lactating mothers practiced Orthodox Christianity. Additionally, over half (54.4%) of the lactating mothers reported that a birth interval >2 years (Table 1).

**Table 1:** Socio-demographic and economic-related characteristics of lactating mothers at Dangila Zuria District Awi Zone, Amhara region, Ethiopia, 2022.

**Background characteristics (*N* = 609)**	**Frequencies**	**Percentage (%)**
**Age of the mother (in years)**		
15–24 years	99	16.3
25–34 years	291	47.8
35–51 years	219	36.0
**Ethnicity**		
Amhara	486	79.8
Awi	123	20.2
**Religion**		
Orthodox	395	64.9
Muslim	125	20.5
Protestant	89	14.6
**Marital status**		
Others (divorced, widowed, and separated)	12	2.0
Married	597	98.0
**Maternal educational status**		
Illiterate	377	61.9
Read and write	104	17.1
Primary school	74	12.2
Secondary and above	54	8.9
**The educational status of the husband**		
Unable to read and write	287	47.6
Able to read and write	159	26.6
Primary	75	12.8
Secondary and above	76	13.0
**Maternal occupation status**		
Housewife	474	77.8
Governmental employee	68	11.2
Merchant	39	6.4
Daily laborers	28	4.6
**The occupational status of the husband**		
Farmer	422	69.8
Merchant	60	10.3
Governmental employee	66	11.3
Daily laborer	49	8.5
**Income (in ETB)**		
<500	34	5.6
501–1,000	92	15.1
1,001–2,000	272	44.7
>2,001	211	34.6
**Family size**		
<3 members	92	15.1
4–6 members	397	65.2
>6 members	120	19.7
**Birth intervals**		
<2 years	278	45.6
≥2 years	331	54.4

### 3.2 Health service utilization and child feeding

Six hundred eight (99.8%) study participants had ANC follow-up. Of them majority of mothers had more than three follow-up visits in the last pregnancy. Almost all lactating mothers didn't suffer from any chronic illnesses during their breastfeeding period.

However, 21 (3.4%) mothers had a history of illness in the last 2 weeks. Nearly all (98.7%) study participants reported that they gave birth at a health facility. All mothers also reported that they have practiced exclusive breastfeeding (Table 2).

**Table 2:** Health service utilization and child feeding of lactating mothers at Dangila Zuria District Awi Zone, Amhara region, Ethiopia, 2022.

**Variables (*n* = 609)**	**Number**	**Percent**
**Number of gravid**		
<2	237	38.9
2–4	259	42.5
>4	113	18.6
**Number of children**		
1–2	246	40.4
3–4	250	41.1
>5	113	18.6
**ANC follow-up in the last pregnancy**		
No	1	0.2
Yes	608	99.8
**Number of ANC visits (*****n*** = **608)**		
One time	41	6.7
Two times	105	17.2
≥3 times	463	76.0
**Counseled about maternal nutrition during your ANC**		
No	3	0.5
Yes	606	99.5
**Do you have any chronic illness**		
No	608	99.8
Yes	1	0.2
**History of illness in the previous 2 weeks**		
No	588	96.6
Yes	21	3.4
**Place of delivery**		
Home	8	1.3
Health facility	601	98.7
**Practice exclusive breastfeeding up to 6 months**		
No	4	0.7
Yes	605	99.3

### 3.3 Dietary practices

The majority of lactating women (83.1%) reported that the main source of their food was their production. Similarly, 502 (82.4%) respondents practice home gardening. The majority of the respondents (71.1%) had a habit of jumping their usual feeding practices. Most of the respondents (88.7%) have eaten additional foods during their lactation period. More than half of the participants (52.1%) reported that they feed more than three times per day.

The majority of the study participants had no history of fasting in the last 24 h. The majority (92.8%) of participants had a habit of eating snacks in the previous 24 h. In general, 204 (33.5%) lactating mothers have good dietary practices (Table 3).

**Table 3:** Source of food and dietary practices among lactating mothers at Dangila Zuria District Awi Zone, Amhara region, Ethiopia, 2022.

**Variables (*n* = 609)**	**Number**	**Percent**
**Have a habit of skipping your usual feeding practice**		
No	176	28.9
Yes	433	71.1
**Eat any additional foods during lactation**		
No	69	11.3
Yes	540	88.7
**Consumption/eating protein-rich foods daily**		
No	163	26.8
Yes	446	73.2
**Consumption/eating fresh vegetables daily**	195	32.0
No	247	40.6
Yes	362	59.4
**Consumption/eating fruits in the last 24 h**		
No	259	42.5
Yes	350	57.5
**Number of meals more than 3 times/in the last 24 h**		
Yes	317	52.1
No	292	47.9
**Do you have a habit of eating snacks in the last 24 h**		
No	43	7.1
Yes	566	92.9
**Do you have a habit of fasting in the last 24 h**		
No	592	97.2
Yes	17	2.8
**Consumption/eating starch foods in the last 24 h**		
No	243	39.9
Yes	366	60.1
**Overall dietary practices**		
Good dietary practice	204	33.5
Poor dietary practice	405	66.5

### 3.4 Dietary diversity

Based on the dietary diversity score of lactating mothers, about three-fourths of the lactating women (73.2%) reported that they consumed grains, white roots and tubers, and plantains in the previous 24 h. Three-hundred sixty-six (60.1%) respondents reported that they consumed pulses (beans, peas, and lentils) in the last 24 h. Similarly, more than half of the lactating mothers consume vitamin A-rich fruits and vegetables in 24 h. Only 139 (22.8%) of the respondents consumed meat, poultry, and fish in the 24 h. In general, 455 (74.7%) lactating mothers have good dietary diversity scores in the previous 24 h (Table 4).

**Table 4:** Dietary diversity among lactating mothers at Dangila Zuria District Awi Zone, Amhara region, Ethiopia, 2022.

**Variables (*n* = 609)**	**Number**	**Percent**
**Grains, white roots, and tubers, plantains in previous 24 h**		
No	163	26.8
Yes	446	73.2
**Pulses (beans, peas, and lentils) in last 24 h**		
No	243	39.9
Yes	366	60.1
**Dark green leafy vegetables in the previous 24 h**		
No	220	36.1
Yes	389	63.9
**Vitamin A-rich fruits and vegetables in the previous 24 h**		
No	270	44.3
Yes	339	55.7
**Other fruits in the previous 24 h**		
No	294	48.3
Yes	315	51.7
**Other vegetables in the previous 24 h**		
No	419	68.8
Yes	190	31.2
**Meat, poultry, and fish in the previous 24 h**		
No	470	77.2
Yes	139	22.8
**Eggs in the previous 24 h**		
No	376	61.7
Yes	233	38.3
**Legumes, nuts, and seeds in the previous 24 h**		
No	382	62.7
Yes	227	37.3
**Milk and milk products in the previous 24 h**		
No	309	50.7
Yes	300	49.3
**Women dietary diversity**		
Inadequate women's dietary diversity	154	25.3
Adequate women's dietary diversity	455	74.7

### 3.5 Food security characteristics

From the 9 HFIAS items; the most frequently reported experience of food insecurity was skipping meals (27.4%) followed by worrying about running out of food (25.8%), and being unable to eat preferred foods (25.1%). Overall, nearly one-third (29.7%) of the lactating mothers were food secure (Table 5).

**Table 5:** HFIAS items among lactating mothers at Dangila Zuria District Awi Zone, Amhara region, Ethiopia, 2022.

**Variable (*n* = 609)**	**Number**	**Percent**
**Worried about running out of food**		
No	452	74.2
Yes	157	25.8
**Unable to eat preferred foods**		
No	456	74.9
Yes	153	25.1
**Eat a limited variety of foods**		
No	491	80.6
Yes	118	19.4
**Eat foods that you did not want to eat**		
No	526	86.4
Yes	83	13.6
**Eat a smaller meal**		
No	517	84.9
Yes	92	15.1
**Skipping meals**		
No	442	72.6
Yes	167	27.4
**No food to eat of any kind in the household**		
No	570	93.6
Yes	39	6.4
**Go to sleep at night hungry**		
No	561	92.1
Yes	48	7.9
**Go a whole day and night without eating anything**		
No	574	94.3
Yes	35	5.7
**Food security status**		
Food secure	181	29.7
Food insecure	428	70.3

### 3.6 Factors associated with lactating women's dietary practice

Table 6 shows a bi-variable and multivariate analysis of factors associated with the dietary practice of lactating mothers. In the Bi-variable level analysis, the mother's occupation, being a daily laborer, was nine times more likely to have low dietary practice than the counterparts (AOR 9.35, 95% CI [8.02, 19.96]). Lactating mothers whose birth interval was <2 years, with family size greater than four members, and food insecure mothers were 2.3, 2.8, and 4.0 times more likely to have low dietary practice than those whose birth interval >24 months, whose family size less than four members and who were food secure, respectively (AOR = 2.3, 95% CI [1.49, 3.56]), (AOR = 2.8, 95% CI [1.65, 4.80]), and (AOR = 4.0, 95% CI [2.63, 6.12]), respectively (Table 6).

**Table 6:** Factors associated with dietary practice among lactating mothers at Dangila Zuria District Awi Zone, Amhara region, Ethiopia, 2022.

**Variables (*n* = 609)**	**Dietary practice**		**COR 95% CI**	**AOR 95% CI**	***P*-value**
	**Good**	**Poor**			
**Age of the mother (in years)**					
15–24 years	33	66	1.00 (0.60, 1.65)	0.67 (0.41, 1.09)	0.261
25–34 years	98	193	0.98 (0.68, 1.43)	0.43 (0.12, 1.49)	0.114
35–51 years	73	146	1	1	
**Maternal educational status**					
Illiterate	139	238	0.59 (0.32, 1.14)	0.74 (0.21, 2.57)	0.236
Read and write	33	71	0.75 (0.36, 1.57)	0.79 (0.22, 2.81)	0.202
Primary school	18	56	1.09 (0.48, 2.44)	0.82 (0.26, 2.5)	0.485
Secondary and above	14	40	1	1	
**The educational status of the husband**					
Unable read & write	113	186	0.53 (0.30, 0.93)	0.45 (0.13, 1.52)	0.608
Able to read and write	53	102	0.62 (0.33, 1.15)	0.35 (0.10, 1.17)	0.207
Primary	19	58	0.98 (0.47, 2.04)	0.95 (0.29, 3.10)	0.490
Secondary and above	19	59	1	1	
**Maternal occupation status**					
Housewife	137	337	1	1	
Gov't employee	34	34	**2.45 (4.21, 30.37)**	0.82 (7.59, 18.33)	0.420
Merchant	10	29	0.84 (0.56, 13.51)	1.90 (0.11, 34.29)	0.460
Daily laborers	23	5	**11.31 (3.99, 44.51)**	**9.35 (8.02, 19.96)**	**0.001**
**The occupation status of the husband**					
Farmer	119	306	1.00	1.00	
Merchant	30	33	**3.00 (1.67, 5.38)**	0.54 (0.16, 1.88)	0.190
Gove employee	27	42	1.28 (0.61, 2.68)	1.79 (0.12, 26.57)	0.851
Daily laborer	28	24	1.81 (0.87, 3.76)	0.22 (0.02, 2.84)	0.760
**Birth Interval of the mother**					
<2 Years	137	194	***2.22 (1.56, 3.16)***	***2.31 (1.49, 3.56)***	***0.004***
≥2 Years	67	211	***1***	***1***	
**Income (in ETB)**					
<500	25	9	0.140 (0.06, 0.31)	0.79 (0.420, 1.496)	0.402
501–1,000	35	57	0.63 (0.37, 1.06)	1.07 (0.702, 1.625)	0.903
1,001–2,000	85	187	0.85 (0.57, 1.27)	0.80 (0.536, 1.201)	0.517
>2,001	59	152	1	1	
**Own agricultural land**					
No	156	249	1	1	
Yes	48	156	0.49 (0.33, 0.72)	0.51 (0.32, 0.83)	0.697
**Family size**					
<3 members	24	68	1	1	
4–6 members	123	274	***2.56 (1.42, 4.61)***	***3.01 (1.56,9.28)***	***0.004***
>6 members	57	63	***2.01 (1.33, 3.05)***	***2.82 (1.65, 4.80)***	***0.001***
**Food security**					
Food secure	107	74	***1***	***1***	
Food insecure	97	331	***4.93 (3.39,7.16)***	***4.03 (2.63, 6.12)***	***0.001***

COR, Crude Odds Ratio; AOR, Adjusted Odds Ratio. The “Bold” indicate that the variable showed that there is a significant association with the out come variable.

## 4 Discussion

Effective dietary practices during lactation are crucial for both maternal health and infant wellbeing. The Essential Nutrition Action (ENA) framework, along with numerous studies, emphasizes the importance of optimal nutrition for lactating women. Adhering to recommended dietary guidelines can enhance maternal health outcomes and support healthy growth and development in children (23). In this community-based cross-sectional study, we measured the proportion of good dietary practices among lactating mothers in the Dangila Zuria Woreda. The findings revealed that 33.5% [95% CI] of lactating mothers practiced good dietary habits during their lactation periods. The finding is consistent with studies conducted in other regions.

A study in Bag Lung District, Nepal, found that 31.8% of lactating mothers had good dietary practices (21), while a study in Wollega showed 33.9%. In Bahir Dar town, 35.9% of mothers demonstrated good dietary habits. Two systematic reviews in Ethiopia reported that 34.3% of lactating mothers c another study in Bahir Dar found that 39.3% of respondents had good dietary practices (10, 12, 20, 36, 37). The similarity may be because of cultural influences, such as shared beliefs about nutrition during lactation, socioeconomic conditions that limit access to diverse foods, common public health initiatives, and educational programs, the use of similar research methodologies, including sampling techniques and dietary assessment tools, and adherence to established nutritional guidelines, like those from the Essential Nutrition Action (ENA), may uniformly influence the dietary habits of mothers in these regions was with this low and similar prevalence.

However, this finding is lower than the study conducted in South Eastern Zone of Tigray 46.7%, in Gondar town 40.1%, rural community of Tigray 43.8%, Addis Ababa 48.6%, and in Dessie town, 45.2% (8, 29, 32–34). This discrepancy may be attributed to variations in regional dietary practices, access to resources, and differences in educational outreach related to nutrition during lactation. Additionally, the studies in these locations may have benefited from more robust health promotion initiatives or differing socioeconomic conditions that support better dietary practices among lactating mothers.

On the other hand, the finding of this study is higher than the study conducted in Samre Woreda, Tigray 28.8%, Finote selam 23.7%, and West Gojjam Zone 19.9%, Mizan Aman Town, Southwest Ethiopia 25.1% (8, 10, 38, 39). This difference may be attributed to geographical variations in agricultural productivity and dietary resources. For instance, the study conducted in Tigray occurred in an area less productive than our study site, which could impact the availability and diversity of food. Additionally, the smaller sample size in the second study may have influenced its findings. Central Ethiopia primarily produces Teff, Dagussa, and cereals, while the northern regions depend more on cereal crops, leading to significant differences in the dietary practices of lactating mothers.

Furthermore, our study found a statistically significant association between mothers' dietary practices and various associated factors, including the mother's occupation, which was specifically linked to the dietary practices of lactating mothers. Lactating mothers who were daily laborers had higher odds of poor dietary practice compared to those who were housewives (AOR = 9.35, 95% CI: [8.02, 19.960]).

This is supported by the studies mountains of Nepal (40), Ambo district Ethiopia (11), and Misha Woreda, South Ethiopia (41). The increased likelihood of poor dietary practices among daily laborer mothers may be attributed to their lower economic status and the demands of their work, which often leave them with limited time and resources to consume the recommended types of nutritious foods. In contrast, housewives may have more opportunities to focus on meal preparation and dietary quality for themselves and their children.

In this study, family size was found to have a statistical association with the dietary practices of lactating mothers. Mothers with a family size greater than four had higher odds of poor dietary practices compared to those with smaller families (AOR = 2.821, 95% CI: 1.656, 4.804). This is supported by studies conducted in Wollega, Guto Gido woreda, and Nekemte referral hospitals (42), East Wollega Zone, Ethiopia (12), as well as a systematic review in Ethiopia (37). The possible explanation for this trend is that women with larger family sizes may become less focused on their dietary practices due to competing demands. When food supply is limited, mothers may prioritize feeding their children or other family members over their own nutritional needs, which can negatively impact both their health and that of their infants.

The finding of this study identified that a mother's birth interval has a strong statistical association with the dietary practices of lactating mothers. Mothers whose birth interval <2 years was had higher odds of poor dietary practice as compared to their counterparts.

This may be due to the increased demands placed on mothers with closely spaced births, as they often prioritize the care of their younger child while managing the needs of the family. Consequently, this can lead to limited time and resources for self-care, making it challenging for them to meet their nutritional needs and those of the entire family. As a result, the focus on immediate child care can significantly hinder their ability to improve their nutritional status.

In this study food security of the mothers has a strong statistical association with dietary practices of lactating mothers. Mothers who had food insecurity had higher odds of poor dietary practice as compared to mothers who were food-secured (AOR = 4.030, 95% CI: 2.653, 6.123). This may have been explained as, when a woman had food security, she became more concerned about good dietary practices and immediately put it into practice. This is supported by a study which was conducted in Boston (43), West Gojam Zone (10), a systematic review in Ethiopia (37), Mizan Aman Town, Southwest Ethiopia (39), food insecurity may worsen diet quality and health of women's in their life.

## 5 Conclusion

In conclusion, this study highlights a concerning prevalence of poor dietary practices among lactating mothers, which is notably lower than findings from other research. The analysis revealed significant associations between dietary diversity and several key factors, including family size exceeding four members, short birth intervals, maternal occupation, and food insecurity. These insights underscore the need for targeted interventions that address these critical areas to improve the nutritional wellbeing of lactating mothers. By focusing on enhancing good dietary practices and addressing economic and social barriers, we can better support mothers in meeting their nutritional needs, ultimately benefiting both their health and that of their children.

### 5.1 Recommendation

Ministry of Health (MOH), Regional Health Bureaus (RHB), and health professionals, should play a critical role in improving maternal nutrition. Specifically, the MOH and RHB should integrate dietary counseling into existing maternal health programs, ensuring that healthcare providers are trained to offer personalized nutrition guidance during antenatal and postnatal visits. This could include incorporating nutrition-focused education into routine health check-ups for lactating mothers, with an emphasis on practical, culturally appropriate dietary practices.

Health professionals, including clinicians, and community health workers, can provide ongoing support through home visits and local health outreach programs. Additionally, these stakeholders should work to strengthen community-based interventions, such as organizing local nutrition workshops and peer support groups, where lactating mothers can learn about balanced diets, food security, and breastfeeding support.

To address food insecurity, the MOH should collaborate with agricultural and social welfare sectors to enhance access to nutritious food in vulnerable areas. Furthermore, promoting women's occupational opportunities and strengthening family planning initiatives, including appropriate birth spacing, will contribute to better maternal health outcomes and allow mothers to focus on optimal nutrition. In general, all stakeholders can significantly enhance maternal dietary practices, improve health outcomes for mothers and children, and foster a healthier future for families and communities.

### 5.2 Limitation of the study

The main limitation of this study is the reliance on maternal self-report through a 24-h dietary recall to assess dietary practices. This method is subject to recall bias, as mothers may not accurately remember or report all foods consumed within the previous 24 h. Factors such as forgetfulness, misunderstanding of portion sizes, or social desirability bias could influence the accuracy of the reported data. Additionally, the dietary assessment does not account for variations in long-term eating patterns or seasonal changes in dietary practices. Future studies could benefit from using more objective measures, such as food diaries or biomarkers, to complement self-reported data and reduce these biases. Furthermore, the cross-sectional design of the study limits the ability to draw causal inferences.

## Data Availability

The original contributions presented in the study are included in the article/supplementary material, further inquiries can be directed to the corresponding author.
